# scFv from Antibody That Mimics gp43 Modulates the Cellular and Humoral Immune Responses during Experimental Paracoccidioidomycosis

**DOI:** 10.1371/journal.pone.0129401

**Published:** 2015-06-19

**Authors:** Grasielle Pereira Jannuzzi, Aldo Henrique F. P. Tavares, Gilberto Hideo Kaihami, José Roberto Fogaça de Almeida, Sandro Rogério de Almeida, Karen Spadari Ferreira

**Affiliations:** 1 Departamento de Análises Clínicas, Faculdade de Ciências Farmacêuticas da Universidade de São Paulo, São Paulo, Brazil; 2 Instituto de Biologia, Universidade de Brasília, Brasília, Brazil; 3 Departamento de Química, Instituto de Química, Universidade de São Paulo, São Paulo, Brazil; 4 Departamento de Ciências Biológicas do Instituto de Ciências Ambientais, Químicas e Farmacêuticas, Universidade Federal de São Paulo, Diadema, Brazil; The University of Texas at San Antonio, UNITED STATES

## Abstract

Paracoccidioidomycosis (PCM), caused by *Paracoccidioides* species is a prevalent systemic and progressive mycosis that occurs in Latin America. It is caused by *Paracoccidioides* species. Immunization with dendritic cells transfected with a plasmid encoding the scFv (pMAC/PS-scFv) that mimics the main antigen of *P*. *brasiliensis* (gp43) confers protection in experimental PCM. DCs link innate and adaptive immunity by recognizing invading pathogens and selecting the type of effector T cell to mediate the immune response. Here, we showed that DC-pMAC/PS-scFv induces the activation of CD4^+^ and CD8^+^ T cells. Moreover, our results demonstrated that BALB/c mice infected with *P*. *brasiliensis* and treated with DC-pMAC/PS-scFv showed the induction of specific IgG production against gp43 and IFN-γ, IL-12 and IL-4 cytokines. Analysis of regional lymph nodes revealed increases in the expression of *clec7a*, *myd88*, *tlr2*, *gata3* and *tbx21*, which are involved in the immune response. Taken together, our results indicate that the scFv modulates the humoral and cellular immune responses and presents epitopes to CD4^+^ and CD8^+^ T cells.

## Introduction

Paracoccidioidomycosis (PCM) is an important mycotic disease in Latin America caused by species of the genus *Paracoccidioides*. Both the *Paracoccidioides brasiliensis (P*. *brasiliensis)* and *P*. *lutzii* species are thermally dimorphic. Infection is initiated through the inhalation of fungal conidia and a primary attack of lung tissues [1].

The innate and adaptive immune responses to PCM have been investigated using several experimental models. The protective immune response has been associated with high levels of IFN-γ and IL-2, while the non-protective immune response has been correlated with increased IL-4 production [2]. Toll-like receptors (TLR) play a role in innate immunity and are crucial to the pathogenic process. Dendritic cells (DCs) are the most effective antigen-presenting cells for inducing cell-mediated immune responses. The interaction between *P*. *brasiliensis* and pulmonary DCs induces IL-10 production and TLR-2 expression, which has been suggested to be a possible mechanism of susceptibility to PCM [3].

The main and most well-characterized antigenic component of *P*. *brasiliensis* is a 43-kDa glycoprotein (gp43) [4]. Previous research has shown that the immunization of mice with anti-gp43 monoclonal antibodies (Ab1) initiates the idiotypic cascade proposed by Jerne and induces both anti-idiotype (anti-Id) (Ab2 α and β) and anti-anti-Id (Ab3) antibody production [5]. The Ab2 mAb 7.B12 inhibits more than 95% of the binding of gp43 to Ab1, suggesting that this mAb represents the internal image of gp43, as proposed by Nesonoff and Lamoyi [6].

In 2004, a T cell proliferative response was demonstrated following the immunization of mice with an Ab2-β Mab (7.B12) and their subsequent exposure to gp43 *in vitro*, indicating that this molecule contains a T cell epitope [7, 8]. However, the use of this antibody can cause damage to the host due to its size and induction of an unspecific immune response.

The peptide P10 of gp43 from *P*. *brasiliensis* modulates the immune response. After P10 plasmid immunization, a stronger protective immune response involving a CD4^+^ T cell epitope has been observed [8]. DNA vaccination has shown therapeutic effects in experimental PCM based on a reduction in fungal burden [9–11].

Previous studies by our group [12] have suggested that a single-chain variable fragment (scFv) that mimics the Ab2-β 7.B12 Mab against gp43 may decrease the yeast cell burden in the lungs of infected mice when it is transfected into dendritic cells (DC-pMAC/PS-scFv). Antibody fragments are currently the most variable proteins that can be employed as therapeutic, diagnostic, and research tools, and they have the largest worldwide market among pharmaceutical proteins [13,14].

The use of transfected DCs carrying inserts is very important in the modulation of infection, and because they have been shown to be efficient in the control of experimental PCM [12], the present study addresses the role of DCs transfected with scFv in modulatory functions in the innate and adaptive immune responses.

Our data further confirm that these transfected cells seem to play an important role during the infection of mice with *P*. *brasiliensis*, as determined by the capacity of the activated immune system. Here, we have shown that pMAC/PS-scFv induces the high expression of CCR7^+^/CD40^+^ molecules in DCs and that the scFv contains epitopes of both CD4^+^ and CD8^+^ T cells, an important aspect in the protection against PCM.

## Materials and Methods

### 1. Mice and ethics statement

Female BALB/c mice (8–12 weeks) were housed under pathogen-free conditions at the animal laboratory facility of the University of São Paulo. The Committee on the Ethics of Animal Experiments of the University of São Paulo approved the protocol in 08/2011 (Permit Number: 321). In the infection experiments, the animals were anesthetized using intraperitoneal ketamine (80–100 mg/kg) in combination with xylazine. The mice were euthanized by carbon dioxide overdose.

### 2. *Paracoccidioides brasiliensis* strain

The yeast forms of the highly virulent *P*. *brasiliensis* strain 18 (Pb18) were grown on Sabouraud agar (Becton, Dickinson and Company, Le Pont de Claix, France) and were used for the infection assays. The viability of the yeast cells was determined using trypan blue. We used cell populations with viabilities of higher than 90%.

### 3. Antigen

The purification of gp43 from an exoantigen of *P*. *brasiliensis* B-339 was prepared as previously described [15]. Gp43 glycoprotein was quantified using the Bradford method [16] and used at a concentration of 20 μg/mL.

### 4. Migratory DCs and T cell profiles

To detect migrating DCs and activated T cells, BALB/c mice were immunized in the thigh by the intramuscular injection of 20μg/mL pMAC/PS-scFv. As a control, we used 20 μg/mL of empty vector (pMAC/PS) or 20 μL of PBS. After 7 days, inguinal and popliteal lymph node cells were obtained and analyzed by flow cytometry with a FACSCanto II (Becton Dickinson). To determine the expression of MHC class II and co-stimulatory molecules in the DCs, we used labeled mAbs against mouse PE CD11c (N418), FITC CD8a (Ly2 53–6.7), FITC DEC-205 (NLDC-145) and PE CD40 (3/23). To determine the T cell profiles, we used labeled Mabs against mouse APC CD3e (145-2C11), PE CD3e (145C11), FITC CD4 (L3T4 6K 1.5), APC CD8a (53–6.7) and PE-Cy5 FoxP3 (FJK-16s) (all antibodies were obtained from BD Biosciences, San Jose, CA). The flow cytometry data were analyzed using FlowJo. Fluorescence-minus-one (FMO) tubes were used as additional controls.

### 5. Gene expression analysis

To analyze the gene expression in inguinal and popliteal lymph node cells, the animals were immunized via the intramuscular route, as described above. After one week, lymph node cells were obtained, and total RNA was extracted using TRIzol (Invitrogen). PCR was performed according to the manufacturer’s instructions. The total RNA was reverse-transcribed to cDNA using RT^2^ qPCR Master Mix (Qiagen Company) and amplified with SYBR Green. PCR was performed with an ABI7500 real-time PCR instrument (Applied Biosystems). The data were analyzed using software version 1.3. The transcription profiles of the following genes were studied: *clec7a*, *gata3*, *tbx21*, *tlr2*, *tlr4* and *myd88*. The *PPC*, *MGDC*, *RTC*, *Gapdh* and *B2m* housekeeping genes were used as assay controls. The calculation was done using 2^-ΔΔCT^ and statistical analysis was conducted on average expression values obtained from three separate experiments.

### 6. T cell epitope prediction

To determine the T cell epitope prediction of the scFv (MTLNMLLGLKWVFFVVFYQGVHCARVKLVESGEGLVKPGGSLKLSCAASGFTFSDFAMSWVRQTPEKRLEWVAYISSAGSYIDYADTVKGRFTISRDNARDTLYLQMTSLKSEDTAIYYCIRDGHYGSTSHWYFDVWGTGTTVTVSSRGGGGSGGGGSGGGGSDLQIVLTQSPAIMSASLGERVTMTCTATSSVSSSYLHWYQQKPGSSPKLWIYSASNLASGVPARFSGSGSGTSYSLTISSMEAEDAATYYCHQYHRSPPTFGGGTKLEIKHHHHHHHGDPKADNKFNKEQQNAFYEILHLPNLNEEQRNGFIQSLKDDPSQSANLLAEAKKLNDAQAQKLKVPSSNSSRPARPDPDMIRYIDEFGQTTTRMQ), we used the Immune Epitope Database Program (IEDB analysis resource). This program provides a collection of tools for the prediction and analysis of immune epitopes. We used a T cell epitope prediction tool that includes MHC class I and II binding predictions, as well as peptide processing predictions.

For this study, we used the following BALB/c mouse alleles: H2-IAd and H2-IEd for MHC class II and H2-Dd, H2-Kd and H2-Ld for MHC class I. The selected MHC class II epitope had an IC50 of <500 nm, corresponding to an intermediate affinity, and the chosen MHC class I epitope had an IC50 of <50 nm, corresponding to a higher affinity.

### 7. Proliferation assay

To evaluate T cell proliferation, inguinal and popliteal lymph node cells were isolated from BALB/c mice after intramuscular immunization with PBS (20μL), empty vector (20 μg/mL) or pMAC/PS-scFv (20 μg/mL). The total cells from lymph nodes (3x10^5^) were cultivated in RPMI 1640 medium containing 10% fetal bovine serum plus 20 μg/mL of gp43 antigen. They were then stained with 5(6)-carboxyfluorescein diacetate *N*-succinimidyl ester (CFSE). After 72 h, lymphoproliferation was analyzed by flow cytometry. Concanavalin (ConA) mitogen was used as a positive control.

### 8. Enrichment of DCs

Bone marrow-derived DCs were generated as previously described [17]. Femurs and tibias were flushed with 3–5 ml of PBS with 1% BSA. Bone marrow cells were allowed to differentiate into DCs via culturing in RPMI (Sigma-Aldrich, St. Louis, MO, USA) supplemented with 10% fetal calf serum (FCS) (Cultilab, Brazil), 10 μg/mL gentamicin (Schering-Plough, USA), and recombinant cytokine GM-CSF (50 ng/mL) (Peprotech, Brazil) for 7 days. On days 3 and 5, non-adherent cells (granulocytes and lymphocytes) were removed, and the media and growth factors were replaced. On day 7, non-adherent cells were removed and analyzed by FACS using DC cell surface markers. More than 90% of the cells were CD11c^+^MHC class II^+^ (data not shown).

### 9. Transfection of DCs with pMAC/PS-scFv

Bone marrow-derived DCs (2x10^5^) were transfected with pMAC/PS-scFv (20 μg/mL) using JetPEI Macrophage (Polyplus Transfection). DCs transfected with an empty vector were used as a control (pMAC/PS). The empty pMAC/PS vector and the vector encoding the scFv were obtained as previously described [12].

### 10. Co-stimulatory molecules in transfected DCs

The effects of DC transfection with pMAC/PS-scFv, the empty vector or the PBS control on DC surface molecule expression were investigated. Phenotypes were analyzed by flow cytometry. To identify the MHC class II and co-stimulatory molecules, labeled Mabs against mouse I-A, CD11c, CD80, CD86 and CD40 were used. FlowJo was used for flow cytometry data analyses, and FMO tubes were used as additional controls.

### 11. BALB/c therapy

Mice were challenged with an intratracheal inoculation of 1x10^6^ Pb18 yeast cells [18]. The mice were divided into four groups, and after 7 and 14 days, they received therapeutic doses of the following via intramuscular injection: 1) PBS only (50 μL); 2) DCs only (1x10^6^ cells); 3) DCs transfected with pMAC/PS (1x10^6^ cells transfected with 20 μg/mL of vector) and 4) DCs transfected with pMAC/PS-scFv (1x10^6^ cells transfected with 20 μg/mL of vector containing scFv). To determine the types of cells activated in the lung, after 21 days of infection, all animals were euthanized. The lungs were removed, and the lung cell phenotypes were examined by flow cytometry. To determine the expression levels of several molecules of interest, we used labeled Mabs against mouse FITC or PE CD11c (N418), FITC CD4 (L3T4 6K 1.5), PE CD3e (1452C11), APC CD8a (53–6.7), PE CCR7 (4B12) and FITC CD40 (3/23) (all antibodies were obtained from BD Bioscience, San Jose, CA). FlowJo was used for analyses of flow cytometry data, and FMO tubes were used as additional controls [19].

### 12. Measurement of cytokines

Cytokine production was analyzed in the lung and regional lymph node, subsequent to the BALB/c therapy. After 21 days of infection, all mice were sacrificed, and the lungs and mediastinal and axillary lymph nodes were removed. The homogenate was filtered in sterile gauze and centrifuged at 1400 x g at 4°C for 10 min, and the supernatants were assayed for IL-12, IL-4 and IFN-γ by ELISA (BD).

### 13. IgG production

Total IgG, and IgG1 and IgG2a isotype production were analyzed at 7 days after the first and second immunizations. Serum samples were obtained via bleeding from the tail vein and stored at -20°C until use. An ELISA for IgG anti-gp43 was performed as previously described, with minor modifications [12]. Briefly, polystyrene microplates (Costar, Cambridge, MA) were coated with gp43 (20 μg/mL), diluted in 0.1 M carbonate-bicarbonate buffer (pH 9.6), incubated at 37°C for 1 h, and stored at 4°C overnight. The plates were washed with phosphate-buffered saline (PBS), blocked with a solution containing PBS, 0.05% gelatin, and 2% bovine serum albumin, and incubated for 1 h at 37°C. After the plates were washed with PBS + 0.1% Tween, 50 μL of different mouse serum dilutions were added and incubated for 2 h at 37°C. The plates were washed, and an anti-mouse peroxidase monoclonal antibody (Southern Biotechnology, Birmingham, AL) diluted 1:25 in PBS + 0.01% gelatin + 0.4% bovine serum albumin was added and incubated for 1 h at 37°C. Horseradish peroxidase-streptavidin (1:1000, Genzyme, Cambridge, MA) was added for 30 min and incubated at 37°C. The reaction was developed with 50 μL tetramethylbenzidine (TMB) (Organon Teknika, the Netherlands) diluted 1:1 with urea peroxide for 10 min. The reaction was stopped with 2 N H_2_SO_4_, and optical densities (OD) were measured at 450 nm with an ELISA reader (Bio-Rad, Hercules, CA). The same steps were followed for the IgG1 and IgG2a subclasses, the result shown is the ratio of IgG2a to IgG1.

### 14. Statistics

Prism 5 (GraphPad Inc.) was used for all statistical analyses. The data were compared using either two-way analysis of variance (ANOVA) followed by Bonferroni’s multiple comparison tests or ANOVA followed by the Tukey-Kramer test [20]. All data are represented as the mean and standard deviation (SD).

## Results

### 1. Immunization with pMAC/PS-scFv results in increases in CD4^+^ T cell and DC numbers in lymph nodes

**Fig 1:**
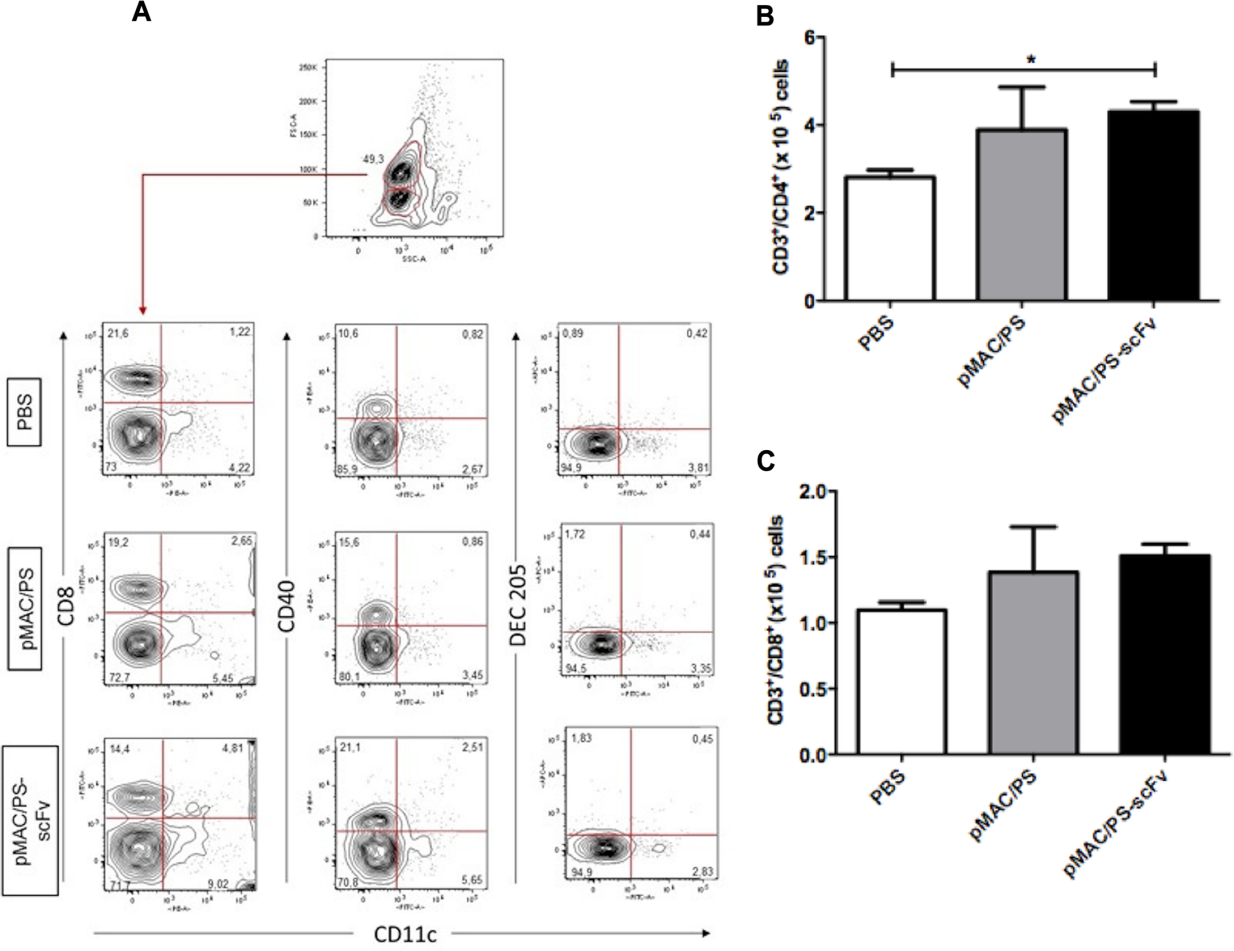
Phenotype of DCs and T cells. BALB/c mice (seven/group) were immunized via intramuscular injection of PBS, pMAC/PS or pMAC/PS-scFv. After 7 days the lymph nodes were obtained and the DCs CD11c^+^/CD8^+^, CD11c^+^/CD40 and CD11c^+^/DEC205 (A) and CD4^+^ (B) and CD8^+^ (C) T cells were analyzed by flow cytometer. *p<0.05. A. Flow cytometry graph: one representative experiment is shown. B and C: Data are expressed as the number of cells obtained with specific Ab. Results are representative of three independent experiments.

T cells and DCs are important in the PCM immune response. To assess the ability of pMAC/PS-scFv to activate these cells, BALB/c mice were immunized with this molecule by the intramuscular route. After a week, total lymph node cells were collected, and the phenotypes were analyzed by flow cytometry. As expected, the mice immunized with pMAC/PS-scFv exhibited increases in CD11c^+^/CD8^+^ and CD11c^+^/CD40^+^ DCs, but we did not observe an alteration in the DEC-205 phenotype (Fig 1A). Therefore, the high expression of CD3e/CD4^+^ was also observed in the pMAC/PS-scFv-immunized mice compared with the PBS-immunized mice (Fig 1B). pMAC/PS-scFv has been shown to induced specific alterations in PCM that are not observed following pMAC/PS treatment [12], although both treatments resulted in the same level of CD3e/CD4^+^ expression (Fig 1B). No significant differences were observed in CD3e/CD8^+^ expression (Fig 1C).

### 2. Analysis of lymph node gene expression profiles after immunization with pMAC/PS-scFv

**Fig 2:**
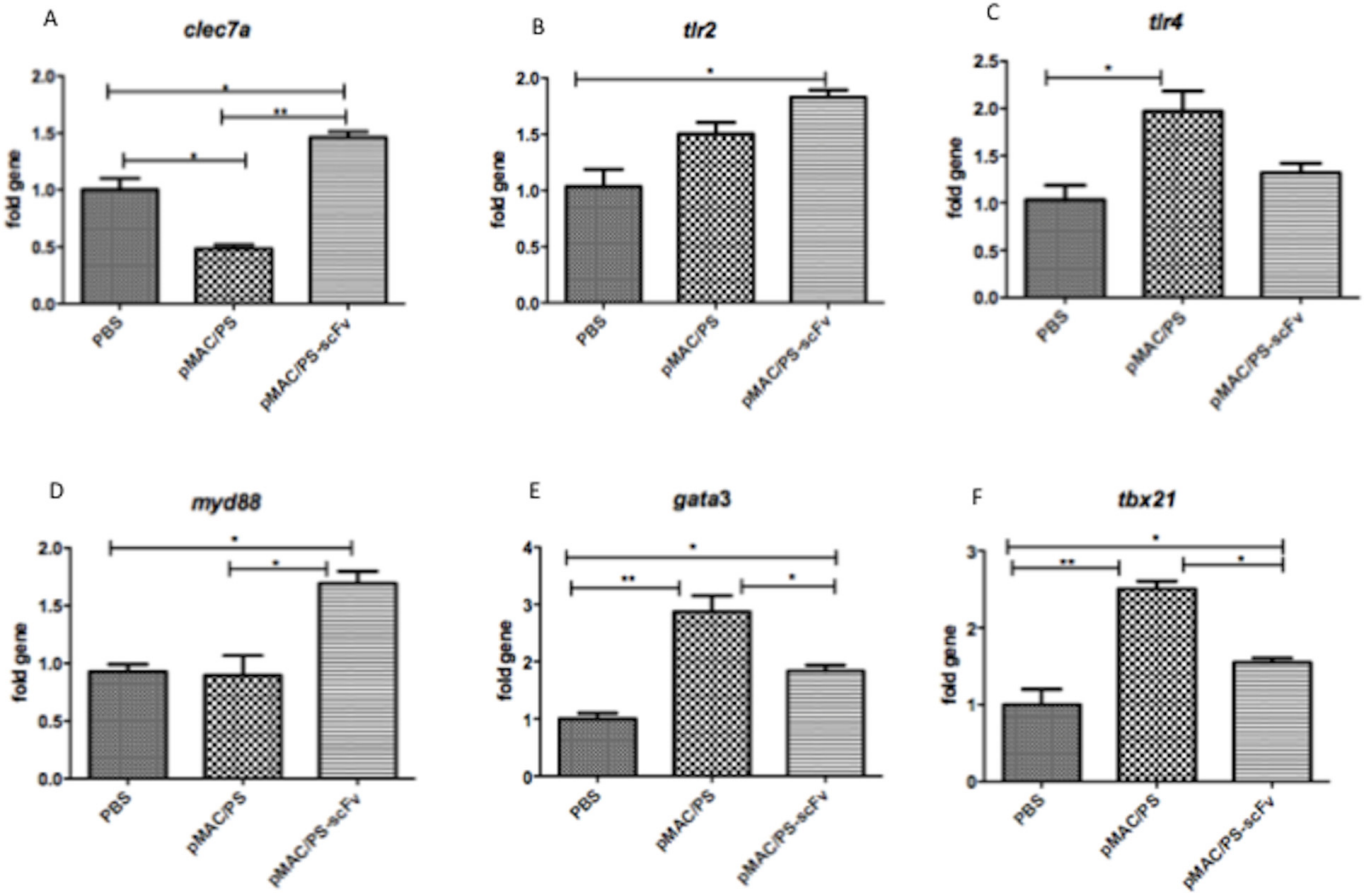
Gene expression. After intramuscular immunization in BALB/c mice (seven/group) with PBS, pMAC/PS or pMAC/PS-scFv, total RNA from lymph nodes was extracted and the gene expression were analyzed by PCR array. (A) *clec7a*; (B) *tlr2*; (C) *tlr4*; (D) *myd88*; (E) *gata3* and (F) *tbx 21*. *p<0.05 and **p<0.001. Results are representative of three independent experiments.

Several genes have been shown to play important roles in the immune response. To determine whether pMAC/PS-scFv alters the expression of these genes, we performed PCR to analyze several genes that are involved in the innate (*tlr2*, *tlr4*, *myd88 and clec7*) and adaptive (*tbx21 and gata3*) immune responses. The results showed significant increases in *clec7*, *gata3*, *myd88*, *tbx21* and *tlr2* expression in regional lymph nodes at 7 days after immunization with pMAC/PS-scFv compared with PBS control immunization. We observed the higher expression of *gata3*, *tbx21* and *tlr4* in the empty vector group compared with the PBS control group. However, when we compared the construct (pMAC/PS-scFv) group with the empty vector (pMAC/PS) group, we observed increases in *clec7a* and *myd88* expression (Fig 2).

### 3. Epitopes to CD4^+^ and CD8^+^ cells

Antigenic peptides displayed by MHC class II and MHC class I molecules are crucial for the activation of CD4^+^ and CD8^+^ cells, respectively. To determine whether pMAC/PS-scFv can be displayed by MHC class I or MHC class II molecules, we analyzed the possible epitopes responsible for activating these cells. The MHC class II H2-IAd binding prediction results revealed the presence of 2 peptides with IC50 values of <500 nm that were 15 amino acids in length (aa): TISSMEAEDAATYYC and ISSMEAEDAATYYCH. In contrast, the MHC class I H2-Kd binding prediction results revealed the following 3 peptides with high affinities (IC50 <50 nm): AYISSAGSYI (10 aa), WYFDVWGTGTTV (12 aa) and SYSLTISSM (9 aa). MHC class II H2 IE-d, MHC class I H2-Dd, and MHC class I H2-Ld showed lower predicted affinities.

**Fig 3:**
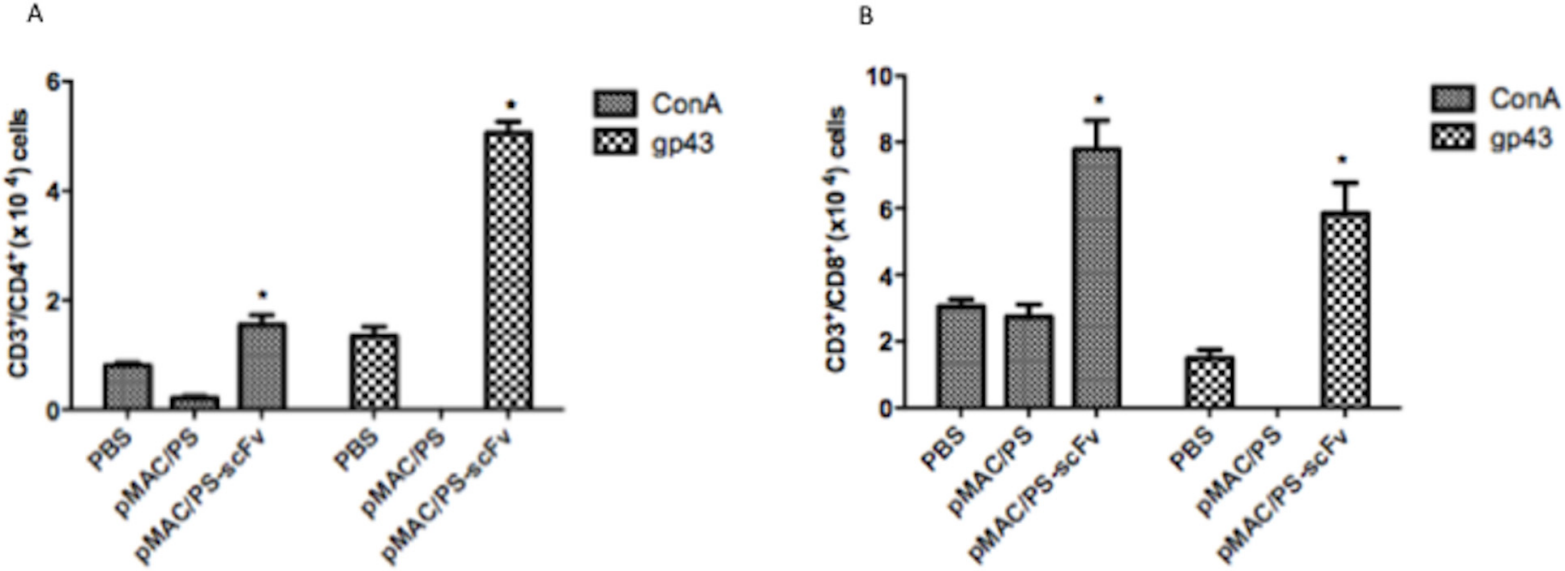
CD4^+^ and CD8^+^ T cells activation. Lymph node cells from seven BALB/c mice per group were obtained after intramuscular immunization with PBS, pMAC/PS or pMAC/PS-scFv. 3x10^5^ cells were cultivated with 20 μg/mL of gp43 antigen. After that, the cells were stained with CFSE and after 72 hours, lymphoproliferation of CD4^+^ (A) and CD8^+^ (B) was analyzed by flow cytometry. As a positive control, we used concanavalin mitogen (ConA). *p<0.05. Results are representative of three independent experiments.

To determine whether these predictions were in accordance with the observed T cell activation, 7 days after intramuscular immunization of the BALB/c mice with pMAC/PS-scFv, we collected regional lymph nodes and cultivated the cells in the presence of gp43. We observed the very specific proliferation of CD4^+^ (Fig 3A) and CD8^+^ T cells (Fig 3B) in the pMAC/PS-scFv-immunized mice compared with the control mice and the pMAC/PS-immunized mice.

### 4. DCs transfected with pMAC/PS-scFv do not show altered molecular expression profiles

**Fig 4:**
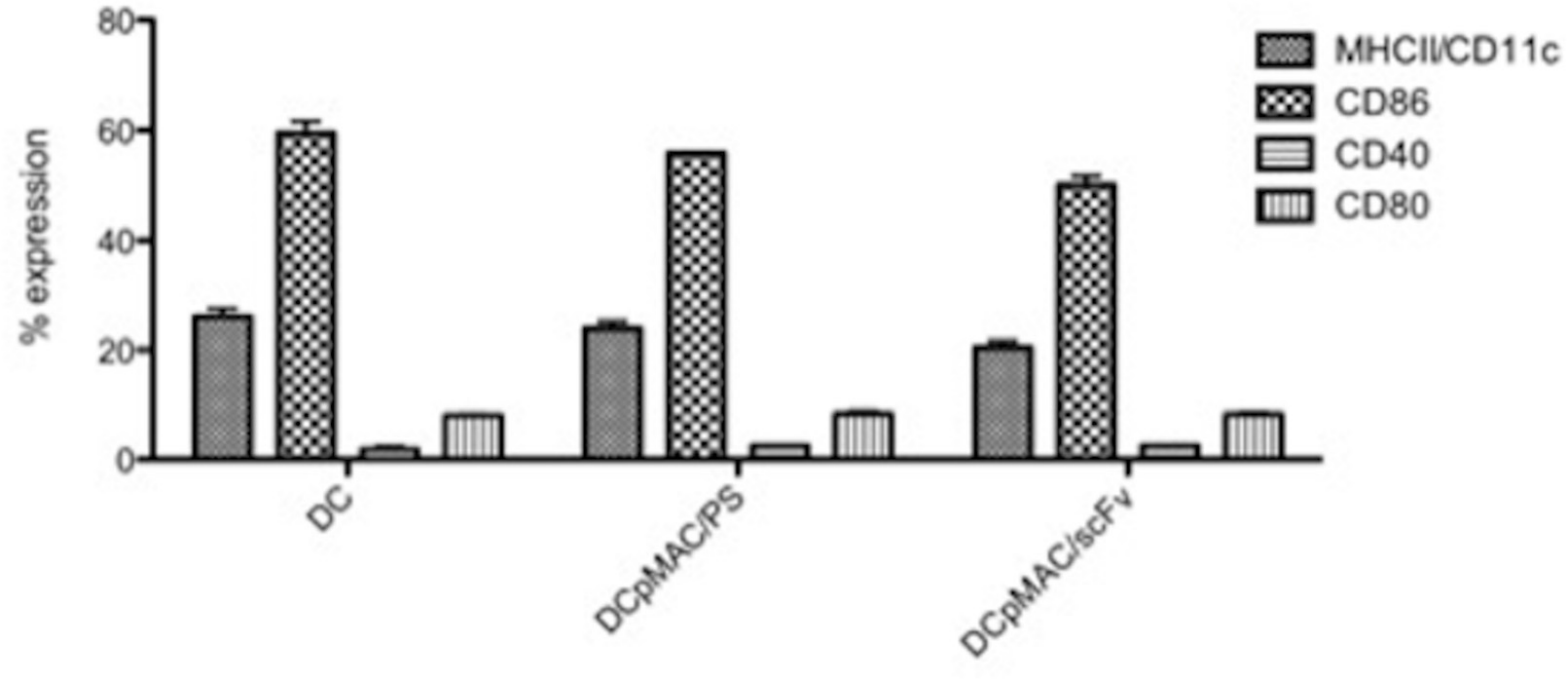
DCs molecule expression. DCs were transfected with pMAC/PS-scFv. After 48 hours the total DCs were stained with anti-MHCII/CD11c, anti-CD86, anti-CD40 and anti-CD80. The data were analyzed by flow cytometer. Results are representative of three independent experiments.

DCs transfected with pMAC/PS-scFv provide important protection against PCM; therefore, we analyzed whether the transfection process could induce the expression of molecules involved in antigen presentation as well as co-stimulatory molecules on the surfaces of DCs. FACS analysis was conducted after 24 h, which was the duration of mouse immunization. At this time point, the DCs were transfected with pMAC/PS-scFv, and the expression levels of MHC class II and co-stimulatory molecules were analyzed. Interestingly, we did not observe any differences in MHC class II, CD80, CD40 or CD86 expression compared with the controls (DCs or DCs transfected with an empty vector) (Fig 4).

### 5. Effects of transfected DCs on T cell activation and migration of DCs

**Fig 5:**
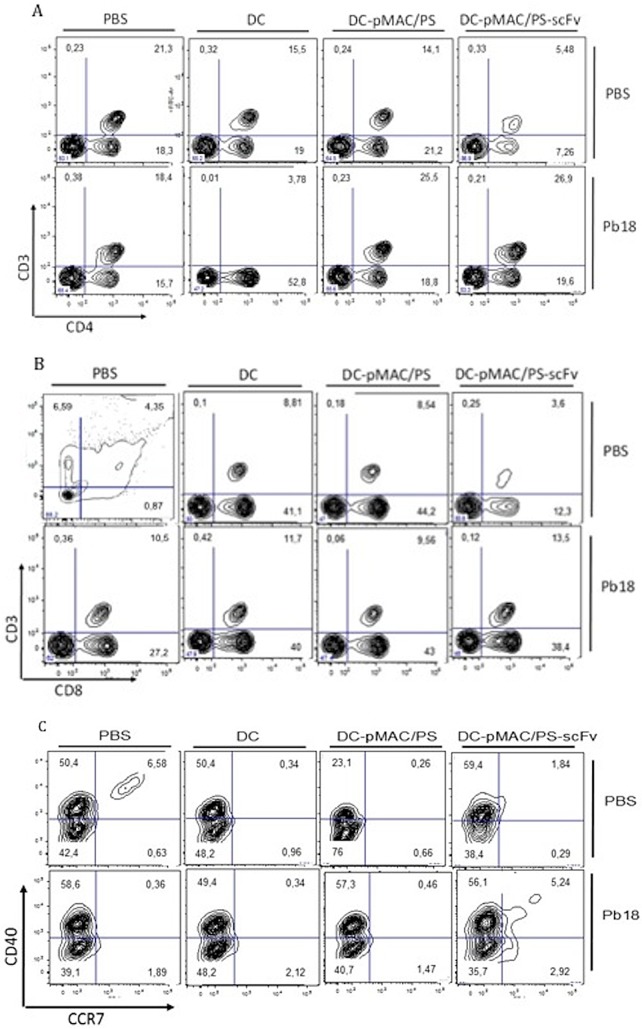
Phenotype of lung cells. BALB/c mice (seven/group) were infected with *P*. *brasiliensis yeast* (1x10^6^). After that, the animals received twice treatments with PBS (50μL), DC (1x10^6^), DC-pMAC/PS (1x10^6^ cells plus 20ng/mL DNA) and DC-pMAC/PS-scFv (1x10^6^ cells plus 20ng/mL DNA). After seven days from the last treatment, the lung cells were stained with anti-CD3e/CD4 (A), anti-CD3e/CD8 (B) and anti-CD40/CCR7 (C). As control of the infection, BALB/c mice received only PBS (50μL) by intratracheal pathway. The data was analyzed by flow cytometer. Flow cytometry graphs: one representative experiment is shown.

To determine whether DCs transfected with pMAC/PS-scFv were capable of stimulating T cells and inducing a migratory DC phenotype, BALB/c mice were intratracheally infected with Pb18 yeast. On days 7 and 14, the animals were treated as described in the Materials and Methods section. At one week after the last treatment, the lungs were collected, and the total cells were labeled with anti-CD3/CD4, anti-CD3/CD8 and anti-CCR7/CD40. As expected, we observed a higher population of CD4^+^ and CD8^+^ T cells after the DC-pMAC/PS-scFv treatment compared with the PBS or DC control treatment (Fig 5A and 5B, respectively); however, no differences were observed in the CD4^+^ or CD8^+^ T cells following the DC-pMAC/PS treatment compared with the DC-pMAC/PS-scFv treatment. Additionally, we observed the increased expression of the migration-related protein CCR7/CD40^+^ (Fig 5C). Although DC-pMAC/PS-scFv did not induce antigen presentation or co-stimulatory molecule expression, a migratory phenotype was observed, suggesting that DC-pMAC/PS-scFv migrated from the lungs to the lymph nodes, where it presented the antigen to T cells through MHC class I and MHC class II molecules.

### 6. DCs transfected with pMAC/PS-scFv modulate cytokine production

**Fig 6:**
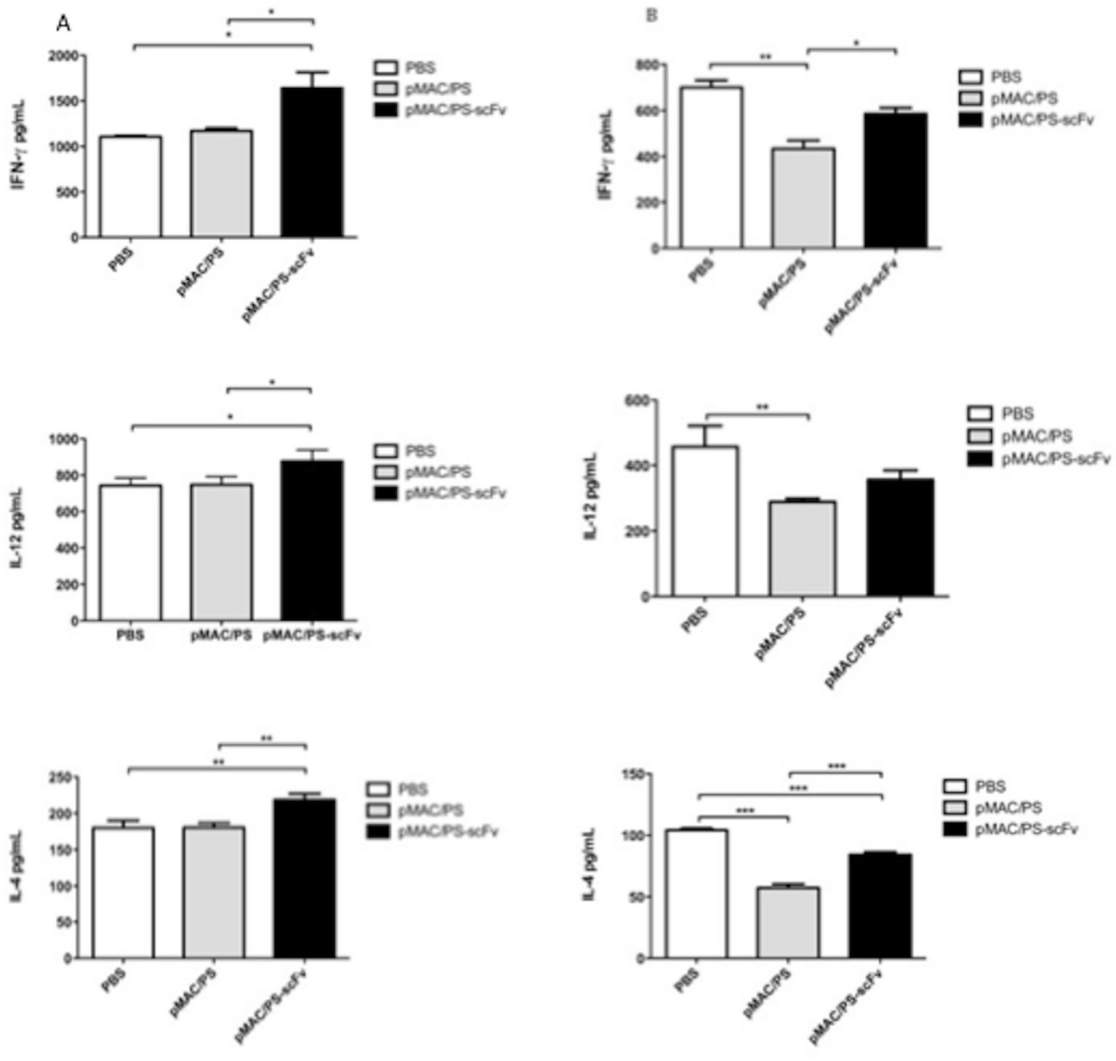
Cytokines. BALB/c mice (seven/group) were infected with 1x10^6^ of *P*. *brasiliensis* yeast. On 7 and 14 days, the animals were treateds with pMAC/PS-scFv. As control, the BALB/c mice was treateds with PBS (50μL) or empty vector. After seven days of last burst, the lung (A) and lynph nodes (B) cells were cultivated in vitro for 24 hours and the IFN-γ, IL-12 and IL-4 were measured by ELISA. *p<0.05 e **p<0,001. Results are representative of three independent experiments.

Cytokines, such as IFN-γ, IL-12 and IL-4, are important proteins during fungal infection. To analyze cytokine production, lymph nodes or lung cells were obtained from untreated controls or BALB/c mice that were previously infected with PB18 yeast and treated with DCs transfected with pMAC/PS-scFv. The cells were cultivated *in vitro* for 24 h, and supernatants were collected and evaluated for IFN-γ, IL-4 and IL-12 cytokine production. Our results indicated significant increases in all of the cytokines analyzed in the lymph node cells (Fig 6A), although these cytokines were down-regulated in the lung cells (Fig 6B) compared with the BALB/c mice that received PBS only or DCs transfected with an empty vector (controls).

### 7. BALB/c therapy with DCs transfected with pMAC-PS-scFv induces IgG2a production

**Fig 7:**
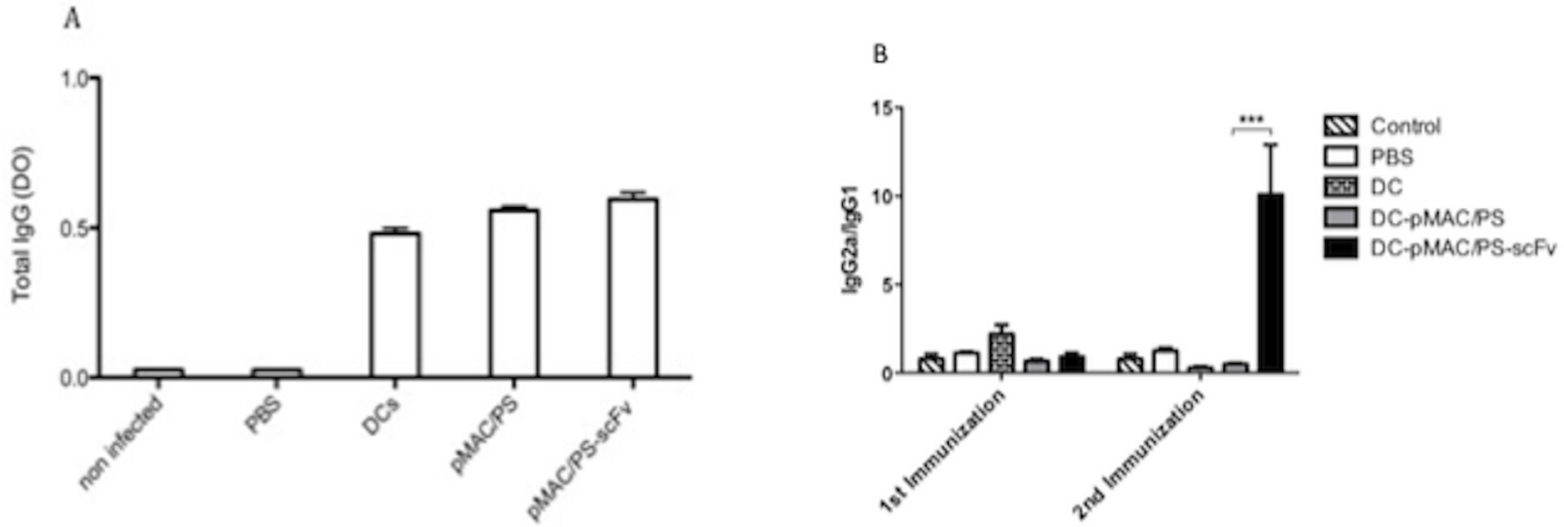
Humoral response. BALB/c mice (seven/group) were infected previously with *P*. *Brasiliensis* yeast (1x10^6^) by intratracheal pathway. The animals were treateds twice with pMAC/PS-scFv and total IgG was measured (A). The ratio between IgG2a/IgG1 was analyse (B) *p<0.05. Results are representative of three independent experiments.

To determine whether DCs transfected with pMAC/PS-scFv induce a humoral response, seven days after each of the treatments, serum samples were obtained from the BALB/c mice and analyzed for the total IgG, IgG1 and IgG2a isotypes. We used a specific antigen (20 μg/mL of gp43) to measure the IgG levels against experimental PCM. We observed an increase in IgG production (Fig 7A) in the sera from the BALB/c mice that received the treatment compared with those in the PBS and control groups. Analysis of the IgG isotypes revealed that the DC-pMAC/PS-scFv treatment induced an increase in IgG2a and a decrease in IgG1 compared with the control treatments (DCs transfected with empty vector or PBS only). A significant increase in the IgG2a/IgG1 ratio was observed in the sera of the mice treated with DC-pMAC/PS-scFv compared with those of the mice treated with DC-pMAC/PS (Fig 7B).

## Discussion

*P*. *brasiliensis* is responsible for one of the most common fungal infections in Latin America. Thus, the need to develop a novel immunotherapeutic model for this infection is of great importance to public health.

In recent decades, various researchers have attempted to develop an anti-PCM therapy, and clinical trials have achieved modest results. Single-chain variable fragment (scFv) antibodies have emerged as an option for passive immunotherapy [21]. J591-scFv, which recognizes the extracellular glycoprotein prostate-specific membrane antigen, has been constructed and can be used in diagnostic and targeted therapeutic applications [22]. KT-scFv is effective against *Candida albicans* rat vaginal infections [23,24]. Additionally, an scFv containing genes encoding capsular polysaccharides provides protection against *Neisseria meningitides* [25]. Therefore, the understanding of the mechanisms of action of scFvs and their modulation of immune function is crucial for developing new therapies. In this investigation, we used an scFv from Ab2-β of the gp43 protein of *P*. *brasiliensis* that confers protection against PCM [12]. We assessed the induction of specific T cell responses. Additionally, we analyzed DCs transfected with this scFv with respect to antigen presentation, cell migration and their ability to efficiently induce a therapeutic effect in experimental PCM.

First, we studied the T cells and molecules activated in lymph nodes from BALB/c mice that were immunized with pMAC/PS-scFv. Our work suggests that APCs are capable of recognizing scFv in the thigh muscle and migrate to regional lymph nodes, where they induce increases in CD11c^+^/CD8^+^ and CD11c^+^/CD40 cells and also stimulate the proliferation of CD4^+^ and CD8^+^ T cells.

CD4^+^ T cells receive a signal from APCs, usually DCs, for activation. CD40L, which is already expressed on activated CD4^+^ T cells, engages with the CD40 that is up-regulated on activated DCs upon PAMP recognition [26,27]. Moreover, CD8α^+^ DCs present antigens on both MHC class II and MHC class I molecules. These cells also have the ability of cross-presentation, whereby an extracellular antigen is taken up and presented on MHC class I molecules to CD8^+^ T cells [28].

These results were confirmed using a lymphoproliferation assay against gp43, a specific antigen of *P*. *brasiliensis*, which showed CD4^+^ and CD8^+^ T cell stimulation. In addition, analysis of the presentation pathways using the IEDB analysis program revealed that the scFv sequence is predicted to stimulate both MHC class II and class I presentation, in agreement with the lymphoproliferation assay. It is important to note that only the DCs transfected with pMAC/PS-scFv was able to stimulate CD8^+^ and CD4^+^ T cell proliferation, demonstrating a specific role of the scFv in contrast with the empty vector. Transfection of the DCs with the empty vector and pMAC/PS-scFv showed similar results. This finding may have been due to the induction of a non-specific immune response by the SV40 tag that is present in scFv construction.

Some studies have shown the importance of CD4^+^ and CD8^+^ T cells in PCM. One such study has shown that both CD4^+^ and CD8^+^ T cells provide protection against pulmonary PCM, but the relative protection of susceptible mice is mainly mediated by CD8^+^ T cells [29].

Considering the importance of the innate and adaptive immune responses in PCM protection, we analyzed several genes known to be involved in recognition, phagocytosis and lymphocyte activation. Our results showed an increase in *gata3* and *tbx21* expression, which could be involved in a variety of Th1/Th2 immune responses. Moreover, we observed increased levels of *clec7a*, *myd88* and *tlr2* gene expression in lymph node cells from immunized BALB/c mice. Taken together, our results suggest that DCs transfected with pMAC/PS-scFv could induce dectin-1, Myd88 and TLR2 expression in the initiation of the innate immune response. Additionally, dectin-1 was able to recognize endogenous ligands in T cells, thereby inducing MHC cross-presentation [30]. Therefore, we postulate that the increase in *clec7a* expression may be involved in CD8^+^ T cell antigen-specific activation through the Myd88 adapter molecule. In addition to TLR2, other TLRs, such as TLR4, have been implicated in the innate recognition of fungal infection [31]. There has been increasing interest in the uses of both Toll/TIR and CLR ligands as adjuvants to enhance vaccine efficacy [32].

The activation of either T cell subset was contingent upon the nature of the fungal therapy, the involvement of distinct innate receptor signaling pathways, and the mode of antigen routing and presentation in the DCs.

DCs are an attractive therapeutic target because they are an essential link between the innate and adaptive immune responses; therefore, we examined the participation of these cells transfected with pMAC/PS-scFv in the control of *P*. *brasiliensis* infection. We have shown that transfected DCs control infection and are responsible for decreasing the fungal burden in the lungs of treated mice [10]. However, the mechanisms involved in PCM protection have been unknown until now.

The transfection process in DCs occurs by endocytosis and can alter surface molecules, but we did not observe a significant difference in surface molecule expression. After being treated with the DCs transfected with pMAC/PS-scFv, the infected mice showed increases in the CCR7/CD40^+^ DC population and in the activation of CD4^+^ and CD8^+^ T cells in the lung. Similar results were observed in lymph node cells from the BALB/c mice immunized by the intramuscular route without transfection. Mouse resistance to PCM is associated with concomitant CD4^+^ and CD8^+^ T cell immunity [29]. In *Aspergillus fumigatus* infection, both CD4^+^ and CD8^+^ T cells mediate vaccine-induced protection from experimental aspergillosis [32]. Moreover, we observed higher CCR7 expression in the DCs, which is necessary for their migration to T cell-populated areas in secondary lymphoid tissues [33] and for efficient signaling to T cells by CD40L [26,27].

T cell activation depends on cytokine production. The cytokine profiles detected in the lung and lymph node cell cultures of the mice treated with pMAC/PS-scFv showed a mixture of Th1/Th2 immune responses. Although the lungs showed decreases in IFN-γ, IL-12 and IL-4, we observed increases in these cytokines in the regional lymph nodes after the treatment. The primary site of PCM is lung tissue; however, after the recognition of the fungus by DCs, these cells migrate to secondary lymphoid organs, where they are activated and induce cytokine production, leading to T cell activation.

The DCs transfected with pMAC/PS-scFv induced a humoral response. We demonstrated specific IgG production against gp43. Interestingly, analysis of the immunoglobulin switch showed an increase in IgG2a after the second treatment but a reduction in IgG1 against the same glycoprotein.

In agreement with our results, Martins and colleagues have demonstrated the predominance of IgG1 over IgG2a in sera collected at 30 days after challenge with radioattenuated yeast of *P*. *brasiliensis* in BALB/c mice. In contrast, in sera collected at 90 days after challenge, the higher production of IgG2a compared with IgG1 has been reported [34].

In conclusion, the present study has revealed that pMAC/PS-scFv immunization or treatments using transfected DCs induce primed CD4^+^ and CD8^+^ T cells *in vivo*. Furthermore, our work suggests that genetic engineering is capable of inducing cross-presentation and modulating the cellular and humoral immune responses that could induce protection to *P*. *brasiliensis*-infected mice.
